# Machine learning and data mining in complex genomic data—a review on the lessons learned in Genetic Analysis Workshop 19

**DOI:** 10.1186/s12863-015-0315-8

**Published:** 2016-02-03

**Authors:** Inke R. König, Jonathan Auerbach, Damian Gola, Elizabeth Held, Emily R. Holzinger, Marc-André Legault, Rui Sun, Nathan Tintle, Hsin-Chou Yang

**Affiliations:** Institut für Medizinische Biometrie und Statistik, Universität zu Lübeck, Universitätsklinikum Schleswig-Holstein, Campus Lübeck, Lübeck, Germany; Department of Statistics, Columbia University, New York, NY 10027 USA; Department of Mathematics, Iowa State University, Ames, IA 50011 USA; Computational and Statistical Genomics Branch, National Human Genome Research Institute, National Institutes of Health, Baltimore, MD 21224 USA; Université de Montréal, Faculty of Medicine, 2900 Chemin de la Tour, Montreal, QC H3T 1N8 Canada; Division of Biostatistics, School of Public Health and Primary Care, the Chinese University of Hong Kong, Shatin, Hong Kong SAR; Department of Mathematics, Statistics and Computer Science, Dordt College, Sioux Center, IA 51250 USA; Institute of Statistical Science, Academia Sinica, Nankang 115, Taipei, Taiwan

## Abstract

In the analysis of current genomic data, application of machine learning and data mining techniques has become more attractive given the rising complexity of the projects. As part of the Genetic Analysis Workshop 19, approaches from this domain were explored, mostly motivated from two starting points. First, assuming an underlying structure in the genomic data, data mining might identify this and thus improve downstream association analyses. Second, computational methods for machine learning need to be developed further to efficiently deal with the current wealth of data.

In the course of discussing results and experiences from the machine learning and data mining approaches, six common messages were extracted. These depict the current state of these approaches in the application to complex genomic data. Although some challenges remain for future studies, important forward steps were taken in the integration of different data types and the evaluation of the evidence. Mining the data for underlying genetic or phenotypic structure and using this information in subsequent analyses proved to be extremely helpful and is likely to become of even greater use with more complex data sets.

## Background

The analysis of complex genomic data is a challenging endeavor that may be tackled using machine learning and data mining techniques. What these methods have in common is that they search through data to look for patterns. To help distinguish machine learning from data mining within this, data mining has been described as the process of extracting useful information from the data. In contrast, machine learning can be viewed as the set of methodological tools to do the extraction [1–3]. Thus, data mining includes selecting, preprocessing and transforming data leading up to the actual application of machine learning methods to build models, which are then interpreted and evaluated. Therefore, machine learning can be seen as 1 specific aspect of a larger class of data mining techniques that focus on algorithms for the automatic recognition of patterns in the data.

As part of the Genetic Analysis Workshop 19 (GAW19), ten groups explored data mining and machine learning techniques. Generally, there were mostly two starting points that motivated these groups. First, many contributors were guided by the data mining idea and assumed that there was an underlying structure in the genomic data which, if identified, could improve inference. For this, the different data types of the GAW data were exploited that included several phenotypic facets, genetic markers, and gene expression data among others. Because the hypothesized structure is not easily accessible as a result of its complexity and limited sample sizes, it is hoped that machine learning methods have the potential to better identify true signals in a lot of noise [4]. The second major motivation was a lack of efficient implementation of machine learning techniques, which was noted as a key conclusion from GAW 15 [5]. Several contributors explored novel approaches that more easily deal with hundreds of thousands of genetic variants at a time [6]. Therefore, the starting points from this group echoed the suggestions by Clark et al [7] to focus first on data mining tools to incorporate prior biological knowledge into data analysis algorithms and, second, on the development of computational methods for machine learning.

In the course of the discussion of our group’s results and experiences, a number of messages were derived. The following summary is structured around these messages. Supporting methods and results will be described along the way.

## Message #1: Using inherent information on data structure helps subsequent analyses

In the spirit of the data mining definition given above, three contributions in our group specifically looked for hidden information in the data to use in further analyses. Of note, this information can be either genetic or phenotypic.

In the contribution by Auerbach et al [8], ancestral information was extracted to potentially improve downstream association analysis with rare variants. They used genome-wide association data from 1851 Mexican Americans with 428,574 single nucleotide variants from odd-numbered chromosomes. Systolic blood pressure (SBP) was the dependent variable. Their method, termed local ancestry summation partition approach (LA-SPA), can be described in the following five steps: First, a principal components analysis (PCA) is performed on the genotypes, and the loading scores of the first component are retained and interpreted to measure global ancestry. Second, residuals are obtained from regressing SBP on age, sex, medication, and the global ancestry measure from the first step. Third, variants are grouped into local, consecutive regions. Fourth, PCA in performed again on the common variants of each local region. This time, the loadings of the first 3 components are used to classify the local ancestral origin of each region for every subject. Specifically, a *K*-means clustering with *K* = 3 is performed on the PCA components, classifying each region of every subject as corresponding to the 3 possible ancestral origins (eg, Caucasian, African, and Native American). Finally, association is analyzed between the residuals from step 2 and the rare variants of each region using a modification of the summation partition approach (SPA) statistic [9]. While the original SPA statistic collapses small groups of rare variants in order to investigate each group’s association with the disease, the following modification considers the association between disease and a region of rare variants, partitioned by the local ancestry of region. The statistic is given by $T={\displaystyle \sum_{k=1}^K}{\displaystyle \sum_{j=1}^J}{n}_{j,k}^2{\left({\overline{Y}}_{j,k}-\overline{Y}\right)}^2$, where *n*_*j,k*_ is the total number of rare variants in region *j* in the *k*th local ancestry, ${\overline{Y}}_{j,k}$ is the mean phenotype of subjects with rare variants in region *j* in the *k*th local ancestry group, and $\overline{Y}$ is the sample average. *P* values are obtained by permutation. Using this method, Auerbach et al [8] tentatively identified associations in regions that would not have been detected by the original SPA alone. Thus, this contribution suggests that including ancestry information improves power for an association test on rare variants.

Yang and Lin [6] also used aspects of the genetic data to identify underlying structure. For this, they focused on homozygosity disequilibrium, which is a pattern of sizable runs of homozygosity that deviates from a random distribution of homozygotes and heterozygotes in the genome [10], and which can be estimated by the homozygosity intensity. In their current contribution, the authors estimate homozygosity intensity and then associate that information with disease phenotypes. In regions with evidence for association, they additionally test for association between gene expression and homozygosity intensity. More specifically, Yang and Lin [6] used the real whole genome sequencing data with 2,769,837 common single nucleotide polymorphisms (SNPs) and 5,578,826 rare variants on the odd-numbered autosomes on 959 related individuals from 20 large pedigrees to estimate the homozygosity intensity for every individual. For this, sliding windows on a chromosome were constructed by using the nearest-neighbor method with a bandwidth of *h*(b), which corresponds to the *b*% of variants on a chromosome that were contained in every window. A double-weight local polynomial model is set up of every individual in every window. For every individual, an estimator of homozygosity intensity in a window centered at physical position of *x*, given by *α*_*0*,_ is then derived by minimizing the locally weighted least squares criterion *E*(*x*) with regard to *α*’s via

$$ E(x)={\displaystyle {\sum}_{i=1}^m}K\left(\frac{x_i-x}{h(b)}\right)L\left({x}_i\right){\left\{{I}_i-\left[{\alpha}_0+{\alpha}_1\left({x}_i-x\right)+\cdots +{\alpha}_p{\left({x}_i-x\right)}^p\right]\right\}}^2, $$

where *m* is the number of variants on the specific chromosome, *p* is the degree of the polynomial, *K*(.) is the kernel weight function defined by $$ K(u)=\left\{\begin{array}{c}\hfill \begin{array}{cc}\hfill {\left(1-{\left|u\right|}^3\right)}^3,\hfill & \hfill \left|u\right|<1\hfill \end{array}\hfill \\ {}\hfill \begin{array}{cc}\hfill 0\hfill & \hfill \mathrm{otherwise}\hfill \end{array}\hfill \end{array}\right. $$, *L*(.) is the locus weight function

$$ L\left({x}_i\right)=\left\{\begin{array}{c}\hfill \begin{array}{cc}\hfill 1,\hfill & \hfill {\mathrm{MAF}}_i\ge 0.05\hfill \end{array}\hfill \\ {}\hfill \begin{array}{cc}\hfill {\mathrm{MAF}}_i/0.05,\hfill & \hfill 0\le {\mathrm{MAF}}_i<0.05\hfill \end{array}\hfill \end{array}\right. $$, and *I* is an indicator taking one if the specific variant is homozygous and 0 otherwise. Thus, variants that are closer to *x* receive higher weight, and the locus weight is designed to reduce the weights of common homozygotes of rare variants, because they carry less homozygosity information. The association of this homozygosity intensity with SBP, diastolic blood pressure (DBP), and hypertension is then modeled within every sliding window using generalized estimating equations in 855 related individuals for whom complete data was available. Finally, in those regions identified in the previous step, generalized estimating equations are again used to model the gene expression from 20,634 transcripts as response with association with homozygosity intensity. An implementation for these analyses is available on the website of the authors (www.stat.sinica.edu.tw/hsinchou/genetics/loh/LOHAS.htm). Applying this approach to the simulation data shows good power and control of the type 1 error. The results by Yang and Lin [6] on the real data demonstrate that the length of regions of homozygosity density differs between individuals, but that there is familial aggregation. The approach identifies interesting genetic regions for DBP and hypertension. In conclusion, information on the genetic structure as defined by homozygosity intensity can be incorporated in the analysis of association with clinical phenotypes and gene expression.

While Auerbach et al [8] and Yang and Lin [6] mine the genetic data for underlying structure, Sun et al [11] exploit additional multiphenotype information to empower subsequent association analyses with rare variants. Their basic idea is to use quantitative blood pressure information to cluster individuals without hypertension (controls), to contrast every cluster with the case sample of hypertensives, and, finally, to combine the association statistics into a weighted sum test. This becomes feasible in the given setting of an unbalanced case-control design, where controls can be split into subgroups to test against the same cases. In more detail, this contribution utilizes data of 42,825 SNPs on chromosome 3 after quality control in 1943 independent individuals with simulated phenotypes. Using a 2-dimensional cluster method on SBP and DBP, the control data is clustered into *K* groups with *K* being determined by cross-validation, so that the resulting *K* groups have different average levels of blood pressure. An association statistic combining the comparison of every cluster with the case group is defined by the clustering sum test (CST) [11], where $CST={\displaystyle {\sum}_{i=1}^K}\frac{d_i}{D}{S}_i$, and *d*_*i*_ is the average phenotypic distance between the *i*^th^ control group and the case group, *D* is the sum of *d*_*i*_, and *S*_*i*_ is the value of a *X*^2^ test statistic contrasting the *i*^th^ control subgroup with the case group, where *i* = 1, …, *K*. Because the case group has the highest level of blood pressure, one hypothesis of CST is that the greater the difference between case and subcontrol group in blood pressure levels is, the more important is the genetic information contained in this subgroup, so that a higher weight should be assigned to that statistic value. Rare variants are accommodated by collapsing them within specified windows into pseudo markers, so that the CST is applied to these pseudo markers. For comparison, the data are also analyzed using the sequence kernel association test (SKAT) [12]. Overall, the number of true positives is higher for CST than SKAT, indicating a greater power of the novel approach. Interestingly, the overlap of identified regions is small, meaning that different signals are picked up by CST and SKAT. It can, therefore, be concluded that using further phenotype information can increase the likelihood of identifying true associations.

In conclusion, the examples from these three contributions show that different data structures may be used creatively to mine a data set for further hidden information, which ultimately may improve subsequent association analyses. Further work is needed to further understand which data structures will be most beneficial in this endeavor.

## Message #2: Exploiting the information from different data types can be successful

For GAW19, we were provided with a realistically complex data set that is comprised of different types of genomic data. This obviously leads to the question of how these different types can be combined in a useful way. A recent review of statistical methods of genomic data integration is available [13]. In our group, two applications used the information from SNPs and gene expression data for association approaches.

As described above, the contribution by Yang and Lin [6] used genotype data in the first step to estimate homozygosity intensity. This was then associated with the clinical phenotypes, and those regions with identified association were finally analyzed for association with gene expression.

In a more direct way, Held et al [14] built support vector machine models to predict disease status from genes that simultaneously collapse genotype variants and use gene expression effects. Specifically, based on 637 individuals with a simulated hypertension phenotype, some or all of the first 150 simulated data sets were used for a selection of interesting genes (training), and three from the remaining 50 simulated data sets were used for classification (testing). For the gene selection step, logistic regression models were fit for every gene as an extension of a previous model by Huang et al [15] with

$$ logit\left(P\left(Y=1\right)\right)=\mathrm{Age}+\mathrm{Sex}+\mathrm{Smoke}+\mathrm{Age}\cdot \mathrm{Sex}+\mathrm{Pedigree}+G+I+G\cdot I. $$

In this model, *Y* indicates the hypertension status, *Sex* and *Smoke* are indicator variables for the proband’s sex and smoking status, *Age* is a continuous measure of the proband’s age, *Pedigree* indicates the proband’s family, *G* is a continuous measure of the specific gene expression, and *I* is an indicator for the presence of any rare alleles at any location in the same specific gene. Thus, for rare variants, this model includes a collapsing that is similar to the original combined multivariate and collapsing (CMC) [16]. Based on each model’s *p* value for testing the null hypothesis that none of the explanatory variables in the model have an effect, interesting genes are then forwarded to the next step of classification where 1 of 3 procedures is applied. In the first, the same logistic regression model as before is applied to the classification data set for the selected genes with separate terms for every gene. In the second and third procedures, support vector machines with radial and linear kernels are used for classification using the same model as in the first step, with additional terms included for the top 1 to 50 genes identified in the first step. The required hyperparameters are derived from cross-validation. Held et al [14] find that the predictive performance is slightly higher for a support vector machine with a linear kernel than for the other methods. With logistic regression and use of a radial kernel, the performance decreases with a greater number of genes. Whether including gene expression information is beneficial for the performance is as yet unclear.

Taken together, these examples show that it is methodologically feasible to exploit genotype and gene expression data either in different steps of the procedure or simultaneously. To what extent these approaches improve results will have to be shown in further experiments.

Another challenge, one that is related to the combination of different data structures, is how to incorporate rare variants effectively when applying machine learning methods. One possibility considered by Auerbach et al [8] is to use information from rare and common variants for different tasks. As described in the previous section, they presume that rare variants contain additional disease-related information that is not present in common variants. Thus, based on common variants, the local ancestry is constructed, and rare variants are then tested for association with the clinical phenotypes. A more general idea was followed by Yang and Lin [6], who use all variants to estimate homozygosity intensity, but common and rare variants receive a different weight to take into account that rare variants carry less homozygosity information.

In a most general approach, rare variants are somehow collapsed into some kind of pseudo markers, as seen in the contributions by Sun et al [11] or Held et al [14]. This could easily be incorporated in other machine learning approaches as well.

Thus, rare variants can be dealt with in machine learning algorithms. Application of collapsing ideas is straightforward but will face similar limitations as in the context of more classical statistical approaches [17]. Furthermore, even though integration of additional sources of data is a promising approach, and machine learning techniques appear adaptable to these settings, ultimate conclusions about which data sources are the best candidates for combination, and best practices about how to combine data are still needed.

## Message #3: Evaluating the evidence from machine learning methods is not straightforward but is possible

In large-scale genomic studies as provided for GAW, we typically analyze the data with two different objectives in mind, which have been contrasted before [5]. The first objective is to identify genetic variants that may play a role, either alone or in concert, in disease development or progression. In contrast, the second objective is to classify or predict disease development or progression based on genetic variants, again either alone or in combination [18]. To meet the latter objective, we have to consider current guidelines for the evaluation of genomic tests like the ACCE framework (www.cdc.gov/genomics/gtesting/ACCE/), which contains criteria for analytical validity, clinical validity, clinical utility, as well as ethical, social, and legal implications [19]. When focusing on the clinical validity, that is, whether a genetic test is able to predict or identify a disease of interest, both the strength of the association and diagnostic or prognostic value have to be measured. As an example for this, the area under the curve was reported in the contribution by Held et al [14].

Most of the contributions of this group, however, followed the first objective of gene identification, for which measures for the strength of evidence are required. Traditionally, *p* values undertake this task. One approach interprets the *p* value as a probability and compares it to an a priori specified significance level, possibly taking multiple testing into account. Based on that comparison, a conclusion regarding the null and alternative hypotheses is reached [20]. This inferential approach is of immediate importance for instance in Phase III clinical trials for regulatory approval, where the decision based on the *p* value may lead to a different action in treating patients, and clear inference about a treatment effect in a defined population is required [21]. In the context of large-scale genomic data, however, instead of methods for inference, we need to rely on methods developed for discovery. Therefore, we use the classical definition of inductive inference by Fisher that allows *p* values to be directly interpreted as measures of evidential strength [22]. Thus, a prespecified significance threshold is not important, and *p* values can be ranked to yield the most promising variants, which may then be taken forward to further validation, functional studies, etc. This approach is also backed up by results from Gorlov et al [23], who showed a linear relationship between the − log *p* value in a discovery study and the chance for reproducibility of the result.

When applying machine learning methods, *p* values are not automatically produced. Oftentimes, though, they may be obtained by permutation. However, this can obviously be time-consuming (see Message #4 below). Another possibility is to utilize alternative measures such as variable importance in random forests [5, 24] or its derivative r2VIM as in the contribution by Holzinger et al [4]. This allows for a more efficient evaluation of evidence by integrating different variable selection parameters. Specifically, Holzinger et al [4] used the whole-exome sequencing data from 1937 unrelated individuals with the simulated SBP and Q1 phenotype. After quality control, 353,103 variants were available. As a benchmark, they derived Bonferroni corrected *p* values from linear regression models, including main effect terms for the variant, medication, smoking, sex, age, and the top 10 principal components. This was contrasted with random forests as implemented in Random Jungle [25, 26] using the r2VIM algorithm that combines the following three components: First, the raw variable importance (VIM) is calculated as the percentage change in mean squared error before and after random variable permutation. Second, the absolute value of the lowest VIM gives an estimate of the null variance and can thus be used as a threshold by selecting only those variables with VIMs greater than this null variance [27]. Here, this estimate is multiplied by a prespecified factor to yield more stringent thresholds. Third, random forests is run several times to select variables that are greater than the specified threshold factor across runs, which is termed *recurrency*. The resulting comparison between linear regression and r2VIM revealed similar true-positive and false-positive frequencies. Thus, it appears that r2VIM is a valid alternative approach when using random forests. However, it is limited by the computational power, as we will discuss in the next section.

## Message #4: Computational limits are still an issue

Computational efficiency was one of the original motivations for applying machine learning and data mining methods. Accordingly, one of the overall messages from our group was that, in practical applications, computational limits are still an issue. This firstly pertains to the analyses themselves, as made explicit in the work by Holzinger et al [4], who found that running random forests in large data sets required a large number of processors provided with a high amount of memory, thus clearly limiting the possibilities for application. In a similar way, other participants restricted their analyses as a result of computational demands in applying artificial neural networks (Legault et al, personal communication). Still others specifically focused on the increase in computational efficiency. Here, Yang and Lin [6] further developed their previous method of estimating homozygosity intensity [28] in that they now circumvent the need for imputing the common homozygote in rare variants, thus decreasing computation time.

As noted previously, computational limits can easily be reached when analyzing interactions between markers [29], because as the number of SNPs increases the number of potential interactions increases exponentially. One possible way to deal with this is to filter promising SNPs before analyzing interactions, an approach which was investigated by Gola and König [30]. Genotype data on 46,746 SNPs in 1829 unrelated individuals after quality control was used with the real phenotypes of SBP, DBP, and hypertension. To select interesting SNPs for later analysis, a collection of filter methods (eg, Relief [31] and subsequent developments) was applied in the first step. These approaches are based on nearest-neighbor techniques by weighting a SNP according to whether the nearest neighbor of the same affection status and the nearest neighbor of the other affection status have the same or different genotypes. These filters are implemented in the multifactor dimensionality reduction (MDR) software package (http://sourceforge.net/projects/mdr/) and can be used on binary phenotypes; in this contribution, they were applied using hypertension as outcome. In the second step, Gola and König [30] then analyzed the selected variants using model-based MDR (MB-MDR) [32] for main effects, interaction with age, interaction with sex, and 2-way SNP-SNP interactions. For comparison, MB-MDR was analogously applied to all unselected SNPs with the phenotypes hypertension, SBP and DBP. Multiple-corrected *p* values were derived by permutation. As a result, interesting nonlinear interaction effects were identified when using the unselected SNP set. However, generally, the filtered SNPs did not match with interesting SNPs from the entire set analysis. Thus, the authors found that the several filtering methods were not suitable in combination with the interaction analysis method, which might be a general problem when using main effects-driven filters for selecting possibly interacting SNPs.

In conclusion, more efficient implementations and more suitable filtering methods are still required for a comprehensive machine learning analysis of genomic data. Although it is acknowledged that computational limits depend directly on the machines, systems, and programming languages used, and that no formal testing of these aspects was performed in our group, this was a recurring theme in our group discussion. It should be noted that computational efficiency is not only relevant in the analyses themselves; it may also be a concern in the process of simulating data, which is described in the next section.

## Message #5: Simulating complex data is a discipline of its own

Machine learning methods are specifically designed to detect or predict structures that are otherwise difficult to find. Even though simulation experiments have strongly been recommended to investigate these methods [1, 27, 33, 34], it automatically follows that these complex structures are also difficult to simulate. Over the past few years, this has become even more of an issue, as complexity relates to different types of genomic data; influence of epigenetic and environmental factors; intermediate and clinical phenotypes; longitudinal, interactive, and conditional effects; and the sheer number of all of these factors. The GAW team has again taken great care to simulate complex phenotypes; however, because different methods are differently tailored to detect different facets of the data, what is simulated might not be what a specific method is designed for. For instance, Holzinger et al [4] hunted for SNP-SNP interactions that were not part of the simulation. Similarly, Held et al [14] investigated the tradeoff of adding more genes to the analysis and the possible benefit of including gene expression data; here, the results may mirror the signal-to-noise ratio and the genotype-gene expression relationship in the simulation, but will not necessarily be conclusive about the performance of the method itself. In contrast to that, the approach by Sun et al [11] included a clustering of control individuals based on their blood pressure levels; this seemed to work well in the simulation data, because the relationship between blood pressure levels, hypertension, and genetic effects had been simulated carefully.

These difficulties emphasize the importance of the development of complex simulation tools. In line with the results from a recent workshop [33], genetic simulation “should not be viewed as simply a useful tool in genetic studies, but should also be viewed as a developing scientific discipline of its own.” Moving forward, there seem to be good reasons to focus on simulating different data sets that might each be appropriate to different scientific questions, instead of a single, highly complex, simulated data set. This could lead to more definitive answers and also be more computationally efficient, though at the potential expense of restricting the meaningfully applicable methods.

## Message #6: Results from complex models are difficult to interpret

Machine learning methods traditionally have been disregarded as “black boxes.” This analogy suggests both that it is not clear what the approach is actually doing, and that their outputs often present a challenge for interpretation [29, 35, 36]. Although this was, again, confirmed in some of our contributions (Legault et al, personal communication) [14, 30], this might not only be a problem of the method but also of the complexity of relationships that can be detected by the method, and of the numbers of variables considered for a specific model. Phrased differently, if classical statistical approaches model similarly complex relationships, the result will also be a challenge for interpretation, and this is more likely to be the case if many features are considered. Thus, regardless of the applied method, one bottom line is that we need to work on improved visualization techniques for complex results (see Gola and König [30] for an example).

On the other hand, the importance of this issue again hinges upon the objective of the study as described above. If we aim at identifying genetic variants that are coresponsible for disease development or progression, interpretability is obviously important. On the other hand, there are good examples where interpretability is sacrificed in favor of model performance. For instance, the Oncotype DX assay is widely used to guide the treatment for patients with different cancer subtypes. This test is based on expression profiling of 21 genes and was proven to be effective to predict the likelihood of cancer recurrence in clinical trials, although lacking a clinical interpretability (eg, Ademuyiwa et al [37] and Cronin et al [38]). Thus, if the aim is classification or prediction of disease or progression, we may accept that a model with excellent prediction properties can be difficult to interpret.

## Discussion

In the course of discussing our machine learning and data mining approaches on GAW19, we extracted 6 common messages. These depict the current state of these approaches in the application to complex genomic data and hint at issues to tackle in future studies.

Additionally, it is worth mentioning that our group applied a number of different machine learning algorithms to the real and simulated GAW data, including random forests, support vector machines neural networks, and MB-MDR. As so often, there was no winner across different scenarios, which is in line with the literature [35, 36, 39]. We can, therefore, confirm that it is usually unclear which method will work best beforehand. On a side note, if different methods fare equally well in terms of classification or prediction, they might have very different looks at the data, which was described as the “Rashomon effect” by Breiman [40] and is an argument for using different approaches to more fully exploit the available information. Further comparisons of single and combined machines are desirable but hampered by the difficulties regarding computational efficiency and available simulation methods as described above. In a similar way, Chen et al [33] concluded that the “…current bottleneck in research is no longer the generation of large-scale genetic data, but the availability of computational tools to effectively analyze the data as well as the means to compare and contrast new tools.”

To conclude, our group found that analyzing the GAW 19 data was a good opportunity to explore different avenues in dealing with complex data using machine learning methods. Although some challenges remain for future studies, important steps were taken forward in the integration of different data types and the evaluation of the evidence. Mining the data for underlying genetic or phenotypic structure and using this information in subsequent analyses proved to be extremely helpful and is likely to become of even greater use with more complex data sets.
